# Methoxyflurane in early analgesic therapy by ski patrol members on Swiss ski slopes – an observational cohort study

**DOI:** 10.1186/s13049-024-01308-9

**Published:** 2024-12-18

**Authors:** Lena Benz, Jürgen Knapp, Fredy-Michel Roten, Markus Huber, Richard Steffen

**Affiliations:** 1 Department of Anaesthesiology and Pain Medicine, Inselspital, Bern University Hospital, University of Bern, Bern, Switzerland; 2 Swiss Air-Rescue (Rega), Zurich, Switzerland; 3 Valais Cantonal Rescue Organisation, Sierre, Valais, Switzerland

**Keywords:** Methoxyflurane, Emergency medicine, Trauma management, Prehospital analgesia, Patient-controlled pain therapy

## Abstract

**Background:**

Pain therapy is an important first-response measure in the pre-clinical care of trauma patients. Injured individuals on ski slopes are usually given first aid by members of the ski patrol. The early implementation of adequate pain therapy by these paramedical rescuers can increase patient satisfaction and have a positive effect on the entire treatment process. In this context, we analysed the administration of methoxyflurane by ski patrol members on Swiss ski slopes.

**Methods:**

In this retrospective observational study, we evaluated 172 datasets, of which 149 concerned patients who were administered methoxyflurane. These datasets were taken from a quality-control survey related to the administration of methoxyflurane by members of the ski patrol in seven ski resorts in the Swiss Alps. The data was collected in the winter months of 2022/23. The ski patrol members had been previously trained by medical professionals and employed methoxyflurane following a defined algorithm, according to which patients with an initial numeric pain score of ≥ 4 qualified for the use of methoxyflurane. After each treatment, data on effectiveness and feasibility were collected by means of a standardised questionnaire. The primary outcome was defined as achieving effective pain therapy, which was designated as a reduction on the numerical rating scale of two or more points and a pain score of seven or less after administration. We then performed a linear regression analysis with the relative pain reduction as the outcome and sex, age, ski resort and injury class as covariates.

**Results:**

Methoxyflurane led to effective pain reduction in around two-thirds of patients on the ski slopes and was easy to use for trained ski patrol members. Median pain reduction was 2 points (interquartile range: 1 to 3) on the NRS scale. The regression model showed lower reduction in pain in lower extremity injuries. Sex, age and initial pain score were not associated with the extent of pain reduction. No serious side effects were observed.

**Conclusion:**

The administration of methoxyflurane by trained ski patrol members is a safe and effective option for early pain management in ski slope injuries. Methoxyflurane could thus represent a useful bridging measure, enabling the ski patrol to relieve moderate to severe pain until professional rescue services arrive. However, it does not seem ideal for lower leg injuries.

**Supplementary Information:**

The online version contains supplementary material available at 10.1186/s13049-024-01308-9.

## Introduction

Every year, around 76,000 people are injured on Swiss ski slopes [1]. Since acute pain is often the most pressing problem for these patients in the first phase of rescue [2], the swift implementation of sufficient pain therapy represents an important prehospital measure as part of initial care, increasing patient comfort and reducing stress [3]. Furthermore, inadequate pain reduction leads to patient dissatisfaction [4, 5]. These patients are usually assessed and initially cared for by qualified ski patrol rescuers [Slope Rescue Service (SRS)].

Given these conditions, the ideal analgesic would have a rapid onset of pain reduction with as few side effects as possible. Moreover, since the users in this situation have only limited medical expertise and, in the first phase of care, are often preoccupied by organisational tasks (securing the accident site, calling for additional rescue personnel and equipment, etc.), the ideal painkiller for use in skiing accidents would not require intravenous access or close monitoring and should be easy to use.

Methoxyflurane fulfils many of these criteria: the effect begins quickly (after several inhalations), the patient can control the administered dosage on their own at any time and the active ingredient is washed out of the body a few minutes after the inhalation has ended [6]. Moreover, its safety and clinical efficacy have already been demonstrated in several large-scale studies [7–10]. Methoxyflurane is an inhalational anaesthetic approved for the emergency treatment of moderate to severe trauma-related pain in conscious adult patients. The specific contraindications include a predisposition to malignant hyperthermia and severe renal and liver insufficiency. The most frequently described side effects are euphoria, sedation, dizziness, cough and nausea.

The aim of the present study was to analyse whether the use of methoxyflurane is practicable in the context of skiing accidents in elevated alpine environments (high altitude, cold, special injury patterns). We analysed the effectiveness in pain reduction on a NRS scale and the satisfaction of users. Additionally, we evaluated the additional demand for painkillers after the initial use of methoxyflurane by the SRS, because this usually determines whether it is necessary to deploy a doctor-manned rescue helicopter.

## Methods

In this retrospective observational cohort study, we examined data from a quality control survey of patients who had been treated with methoxyflurane by the SRS.

### Study design

Data collection took place in seven ski areas in the Swiss Alps (Zermatt, Saas Fee, Saas Grund, Grächen, Bellwald, Belalp and Leukerbad). The participating members of the SRS received structured training in the use of methoxyflurane by the responsible medical director, who provided detailed instruction concerning the indications, contraindications, possible side effects and patient instructions for correct use. The medical responsibility for delegating medical competencies to the members of SRS lay with the medical directors of the individual ski areas. The rescuers were free to use or to refrain from using methoxyflurane at any time. The methoxyflurane had to be administered according to a strict algorithm (Supplement 1). This study describes the results of the quality survey conducted in the winter months of 2022/2023. The evaluation of the coded datasets was approved by the responsible ethics committee of the canton of Bern, Switzerland (2024 − 00611).

Methoxyflurane was employed in the form of prepared application sets (PENTHROX liq 99.9%, Future Health Pharma GmbH, Wetzikon, Switzerland). A maximum of one dose (3 mL) was administered per patient, and the dosage was not repeated. Patients with a pain intensity of four or more on the Numeric Rating Scale (NRS) were eligible for administration. Methoxyflurane was not used if the patient refused pain therapy, if there were changes in consciousness (GCS < 15, e.g. due to craniocerebral trauma, intoxication, etc.) or if there was hemodynamic instability. Further exclusion criteria were known severe kidney or liver diseases, age < 18 years, possible pregnancy or a family history of malignant hyperthermia. For safety reasons, further use was not permitted during helicopter transport, as the responsible authorities have not issued clear guidelines regarding the administration of volatile anaesthetics inside a helicopter cabin. Accordingly, the methoxyflurane therapy was terminated when the patient was turned over to the HEMS crew. Sufficient pain reduction is described in the literature as a reduction of ≥ 2 points or > 25% of the initial NRS [6]. However, since an NRS of ≥ 8 points is generally defined as severe pain [7], we decided to categorize analgesia as ‘effective’ if the reduction was ≥ 2 points *and* the absolute value after treatment was ≤ 7. The additional use or continuation of pain therapy by intravenous medication during transport was left to the discretion of the HEMS crew. In this text, we have described analgesic therapy as ‘sufficient’ only if no additional medication was necessary.

To evaluate the influence of the use of methoxyflurane on the deployment tactics for slope rescue, we had to evaluate the ‘Zermatt’ ski resort separately from all the others. Since Zermatt occupies a distinctive geographical location with very long ground-based transport routes to the nearest hospital (more than one hour, from the valley stations of the mountain railways in Zermatt), helicopter transport (which takes around 20 min) is used almost exclusively there regardless of the urgency and severity of the injury. By contrast, in the other ski resorts, a rescue helicopter is only alerted if sufficient pain relief is not possible with the measures available to the SRS.

### Data collection

After each use of methoxyflurane, the SRS had to fill out a printed form (Supplement 3), which was primarily intended to enable quality control. These forms were then gathered together, and the data was transferred to a single data sheet. In terms of patient-specific data, the forms only contained initials, sex and date of birth. If methoxyflurane was not used, the corresponding contraindication was noted. In addition to the pain-relieving effect [pain score (NRS) when the SRS arrived and when the patient was handed over to the further treatment teams], possible side effects were systematically recorded. Moreover, the general satisfaction of the SRS and any problems with the treatment and handling (weather conditions, preparation of the pipe, problems with the treatment algorithm, etc.) were surveyed. In patients who were referred to the HEMS teams for further treatment, information on additional analgesic therapy (medication used and corresponding dosages) could be evaluated from the HEMS protocols.

### Statistical analysis

Patient demographics were presented in a table. Continuous variables were summarised by mean and standard deviation if normally distributed, or by median and interquartile range if skewed. Categorical variables were summarised with counts and percentages for each level of variables.

To identify the factors influencing the effectiveness of methoxyflurane, a multiple linear regression model of relative pain reduction (change in NRS divided by maximal NRS) against the covariates sex, age, location (ski resort) and type of injury was used. The influence of the type of injury on the effectiveness of methoxyflurane was assessed by means of a likelihood ratio test comparing two linear regression models: one featuring the type of injury as a covariate and the other without the type of injury. To more clearly visualise the influence of different injury types, the estimated marginal means of reduction in pain score were calculated per category.

## Results

A total of 172 questionnaires were completed during the observation period. Methoxyflurane was used in 149 patients (53% male, median age 48 years). Documented reasons for non-use included: age < 18 years (15 patients, 9%), altered consciousness with GCS < 15 (4 patients, 2%), patient rejection (3 patients, 2%) and circulatory instability (1 case, 1%) (Table 1, Supplement 2).

**Table 1:** Baseline characteristics

**Variable**		
Total *N* = 149		N
**Demographics**		
age in years [IQR]	48 [30;59]	147
sex		141
female	62 (44.0%)	
male	79 (56.0%)	
location		149
ski resort ‘Zermatt’	53 (35.6%)	
ski resort ‘Saas-Fee’	63 (42.3%)	
other ski resorts	33 (22.1%)	
injury class		67
trunk	9 (13.4%)	
lower arm	5 (7.5%)	
lower leg	13 (19.4%)	
upper arm	27 (40.3%)	
upper leg	13 (19.4%)	
**Primary outcome**		
NRS max [IQR]	8 [7;9]	149
NRS reduction [IQR]	-2 [-3;-1]	130
effective pain reduction:		130
NO	54 (41.5%)	
YES	76 (58.5%)	

**Fig. 1:**
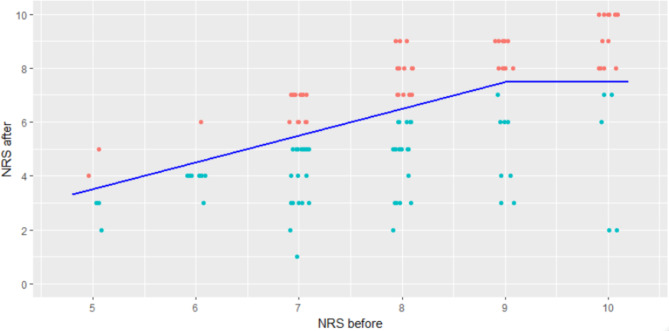
Pain scores before and after treatment using methoxyflurane. Blue line = limit for effective pain therapy, defined as a reduction in the NRS score by ≥ 2 points *and* NRS ≤ 7 after application, red = ineffective pain reduction, cyan = effective pain therapy; NRS = numeric rating scale

**Fig. 2:**
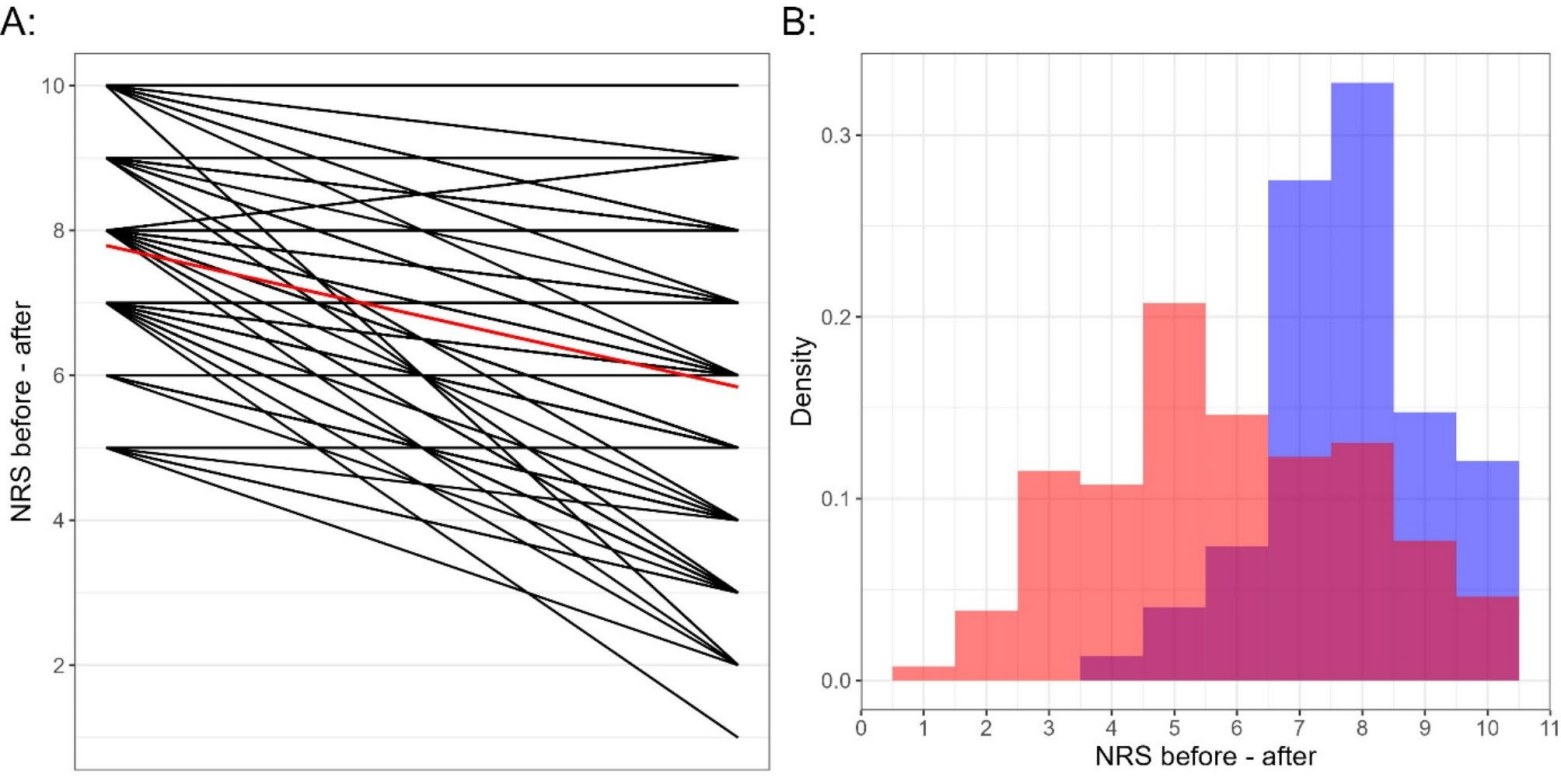
Pain scores of the entire study population before and after the application of methoxyflurane. **A** representation of the before and after NRS for each individual, red = median pain scores of all patients; **B** relative distribution of pain scores, blue = initial NRS, red = NRS after application of methoxyflurane; NRS = numeric rating score

The initial pain score was a median of 8 points on the NRS (IQR 7 to 9). The median reduction in pain was 2 points (IQR − 3 to -1). Moreover, in 59% (95% CI: 49–67%) of all patients, effective pain reduction (pain reduction ≥ 2 points on NRS *and* NRS after treatment ≤ 7 points) was achieved (Figs. 1 and 2).

**Fig. 3:**
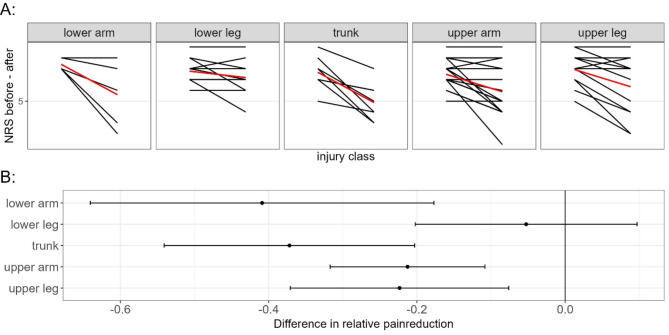
Analysis of the effectiveness of methoxyflurane in relation to the corresponding injury pattern. **A** before and after graphic of the NRS per body region (red = median pain scores); **B** Difference in relative pain reduction per injury class including lower and upper confidence interval (Estimated Marginal Means (EMM))

A multiple regression analysis showed that sex, initial NRS and age were not associated with pain reduction (Table 2), but that the analgesic effect was worse in lower extremity injuries (median difference in pain score: 0 points, IQR 0 to -1) (Fig. 3). The likelihood ratio test comparing the models with and without the variable ‘injury class’ resulted in a p-value (Pr(> Chi)) of 0.03. This indicates that the inclusion of ‘injury class’ in the model significantly improves the fit of the model.

**Table 2:** Multivariate Linear regression models on relative Pain reduction

Characteristic	Beta	95% CI^1^	*p*-value
sex			
female	—	—	
male	-0.06	-0.20, 0.09	0.4
age	0.00	-0.01, 0.00	0.045
location of injury			
ski resort ‘Zermatt’	—	—	
ski resort ‘Saas-Fee’	0.05	-0.11, 0.21	0.5
other ski resorts	0.03	-0.13, 0.19	0.7
injury class			
trunk	—	—	
lower arm	-0.04	-0.32, 0.25	0.8
lower leg	0.32	0.09, 0.55	0.008
upper arm	0.16	-0.04, 0.36	0.1
upper leg	0.15	-0.09, 0.38	0.2

^1^CI = Confidence Interval

As described in the methodology section, there were clear differences in patient transport strategies between the different ski resorts. As expected, more patients were transported by helicopter in Zermatt (34 of 53 patients, 64%). Of these patients, 28 (53% of all patients from Zermatt) received additional analgesic therapy from the HEMS crew. In contrast, 33 out of a total of 96 patients (34%) from the other ski areas were transported by HEMS. Of these patients, 25 (26%) received further analgesic therapy. A total of 67 patients were transported by HEMS. Of these patients, 37 received fentanyl alone, 6 patients were treated with ketamine only and 10 patients received both. The median dose of fentanyl was 100 µg and that of ketamine was 75 mg.

Minor problems during use of methoxyflurane use were described in 32 patients (22% of applications): in 21 patients (14%), users reported difficulties in preparing and administering the medication due to the weather or other external influences, which included specific aggravating conditions, such as extreme cold and wind, steep terrain or extreme sunlight. Technical problems with preparation were described in 10 patients (7%), especially difficulties filling the inhaler with medication while wearing gloves. In one case (1%), language problems were reported as a complicating factor when instructing the patient to inhale the medication. No serious side effects were reported, but moderate side effects occurred in 36 patients (25% of applications): drowsiness (13, 9%), euphoria (13, 9%), dizziness (7, 5%), nausea (2, 1%), coughing (1, 1%) and a ‘gloomy mood’ (1, 1%). The users were ‘very satisfied/satisfied’ in 67% of the applications, ‘moderately satisfied/moderately dissatisfied’ in 24% and ‘dissatisfied/very dissatisfied’ in 9%.

## Discussion

Our study was able to show that methoxyflurane can be used safely and effectively even under these highly specific circumstances. The ski patrol members were able to implement early and sufficient pain therapy for 59% of the injured patients in this study, without any need for additional pain medication and without any significant problems with handling and administration.

In patients with lower leg injuries, however, methoxyflurane seems to have insufficient pain-relieving effect. As a result, 10 out of 13 patients with this injury pattern required additional analgesic therapy by HEMS. From a practical point of view, this result could potentially be explained by the fact that this type of injury frequently requires movement of the injured part during splinting and packaging with an almost inevitable increase in pain, which may not be as significant in other body regions (e.g. shoulder, femur, forearm).

With the exception of the remote area of Zermatt, methoxyflurane alone was sufficient in two-thirds of patients at the ski resorts, meaning that no HEMS had to be called on to provide additional pain therapy. The patients could be transported to the valley by sledge and cable car and handed over to the EMS for transport to the hospital or general practitioner’s office without additional pain medication. If we only look at patients outside of the ski area of Zermatt, only 25 of 96 patients (26%) required any additional analgesic therapy from the HEMS teams.

In the entire study population (including Zermatt patients), 67 received additional analgesic therapy by HEMS. Due to the stated ban on administering methoxyflurane in the helicopter cabin, all patients’ inhalative therapy was terminated when they were handed over to the HEMS teams. The transport time from the ski areas to the nearest hospital was more than ten minutes in every case. Due to the rapid pharmacokinetics of methoxyflurane, it can be assumed that the analgesic effect was completely washed out during this time. This is certainly one possible reason for why additional intravenous pain medication was required. In addition, in half of these patients, the dosage required to achieve what the HEMS crew considered to be sufficient therapy was very low, amounting to only ≤ 100 µg fentanyl, with no need for ketamine. Only 32 (21%) of all patients in this study required higher doses of additional acute pain medication (fentanyl > 100 µg or ketamine at any dosage).

The side effects reported were all moderate and corresponded to those already described. No serious incidents occurred. This is consistent with the findings of several previous studies describing the safety of methoxyflurane [8–12].

Other studies that investigated therapeutic approaches to analgesic therapy without intravenous access (e.g. nalbuphine nasal or fentanyl buccal) [13, 14] showed better pain reduction (3 points on the NRS) as compared to methoxyflurane in this study. However, the clinical relevance of this difference is difficult to assess. The advantages of methoxyflurane compared to these drugs include the fact that the analgesic therapy is always patient controlled, and the analgesic effect can be easily controlled due to the rapid pharmacokinetics. Nasal administration can be more difficult for patients on the ski slopes due to cold-induced vasoconstriction of the nasal vessels leading to altered absorption or to a stuffy nose. Opiates carry a significant risk of respiratory depression. While this side effect was never described with methoxyflurane in the studies cited, it is listed in the drug descriptions of transmucosal fentanyl as ‘occasionally’ (i.e. in 1/1000 to 1/100 cases), which has also been confirmed in a study by Wedmore [15] on the battlefield. Here, one case of a severe respiratory depressant effect was reported in 286 applications. In addition, opioids are subject to very strict legal regulations in many places, which can make it more difficult to dispense them to ski patrol members. In contrast, disadvantages of methoxyflurane are higher costs and more complex preparation: The price of a methoxyflurane inhaler is about twice as high as that of a dose of buccal fentanyl or intranasal nalbuphine. While fentanyl for buccal use is available as a ready-to-use “lollipop”, the methoxyflurane inhaler must be filled manually before use. Egger et al. described the use of methoxyflurane in the Tyrolean mountains in their ‘PainDrop’ study. The use was protocolled in 20 patients, so the subgroups for the individual injury patterns were very small, resulting in limitations regarding the generalisability of the effectiveness per body region. The external influences also differed clearly compared to our study: the survey was carried out primarily in the summer months at temperatures of around 16 °C and at a moderate altitude of a median of 1,910 m above sea level. The study included mountain bikers who had suffered accidents, so it can be assumed that there were differences in the injury patterns of our patients, as well as the terrestrial conditions. Nevertheless, the authors arrived at comparable results: the initial pain score was 7.2 points, with a mean pain reduction of 2.9 points. In this study, too, no serious side effects occurred [16].

To our knowledge, this is the first study to analyse the effectiveness of methoxyflurane at high altitudes (in this study, it was used at up to 3,883 m above sea level) and cold temperatures. Windsor et al. postulated that the drop in outside temperature could have an influence on the saturated vapour pressure and thus the evaporation of volatile anesthetics [17]. However, we were able to show that the effectiveness of methoxyflurane is maintained even under these conditions.

Of course, our study does have limitations. First, it is based on a quality assurance study on the use of methoxyflurane. The patients were not followed up after they were transferred to the emergency room, and the datasets are partially incomplete. Thus, for example, the injury pattern was only correctly documented in about half of the applications, leading to small group sizes for the individual injury categories and limiting the concrete conclusions. Nevertheless, using the available dataset, we were able, for the first time, to assess the pain-relieving effect in relation to detailed body regions in patients on ski slopes. Second, the pain values were recorded directly by the SRS, who also gave the indication for use and the administration of methoxyflurane. There may have been a distortion in the assessment of the initial pain intensity due to suggestive influences from the SRS, if they were already convinced of or sceptical about the effectiveness of methoxyflurane. However, the chosen definition of effective therapy (reduction of NRS ≥ 2 and NRS after application ≤ 7) can still clearly discern clinical effectiveness. Third, the exact geographical and meteorological conditions were not recorded. This means that a statement about the effectiveness of methoxyflurane at high altitudes or at low outside temperatures is only possible to a limited extent. The ski areas studied are located at altitudes between approx. 1,300 and 3,883 m above sea level, and no relevant difference in effectiveness could be shown between the individual locations. By recording all applications within the defined period, a representative sample can still be assumed and the good applicability of methoxyflurane under these circumstances can be postulated.

## Conclusion

The administration of methoxyflurane by trained ski patrol members is a safe and effective option for early pain management in the case of ski slope injuries. The side effects were few, not serious and consistent with what is described in the literature. The use of methoxyflurane could thus represent a useful bridging measure, enabling the relief of moderate to severe pain until professional rescue services arrive. However, it does not seem to be ideal for lower leg injuries resulting from skiing accidents.

## Electronic supplementary material

Below is the link to the electronic supplementary material.

Supplementary Material 1: Including algorithm for the administration of methoxyflurane. NRS = numerical rating scale, GCS = Glasgow Coma Scale, TBI = traumatic brain injury, SOP = standard operating procedure.

Supplementary Material 2: Data collection form for the use of methoxyflurane in the participating ski areas.

Supplementary Material 3: Flow chart of patient distribution.

## Data Availability

The datasets used and/or analysed during the current study are available from the corresponding author on reasonable request.

## Abbreviations

CI: Confidence Interval
EMM: Estimated Marginal Means
HEMS: Helicopter Emergency Medical Service
IQR: Interquartile Range
NRS: Numeric Rating Scale
SRS: Slope Rescue Service
