# Supplementary figures and images for: Methoxyflurane in early analgesic therapy by ski patrol members on Swiss ski slopes – an observational cohort study
Source: Scand J Trauma Resusc Emerg Med. 2024 Dec 18;32:132. doi: 10.1186/s13049-024-01308-9 (PMC11653995; doi:10.1186/s13049-024-01308-9)

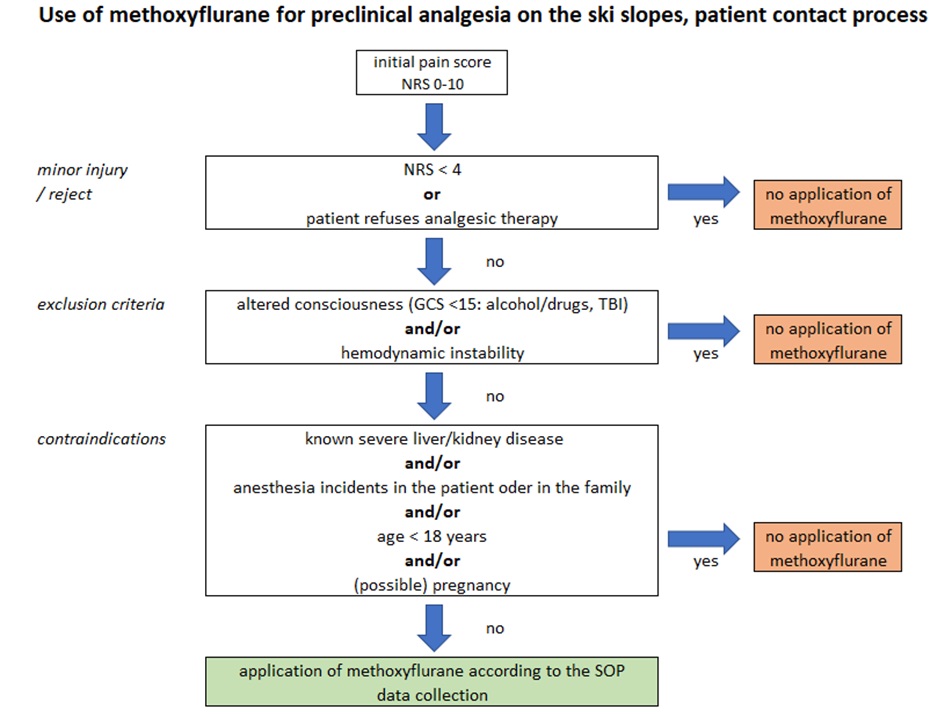

Supplement: Supplementary file 1 — Supplementary Material 1: Including algorithm for the administration of methoxyflurane. NRS = numerical rating scale, GCS = Glasgow Coma Scale, TBI = traumatic brain injury, SOP = standard operating procedure. [file 13049_2024_1308_MOESM1_ESM.jpg]

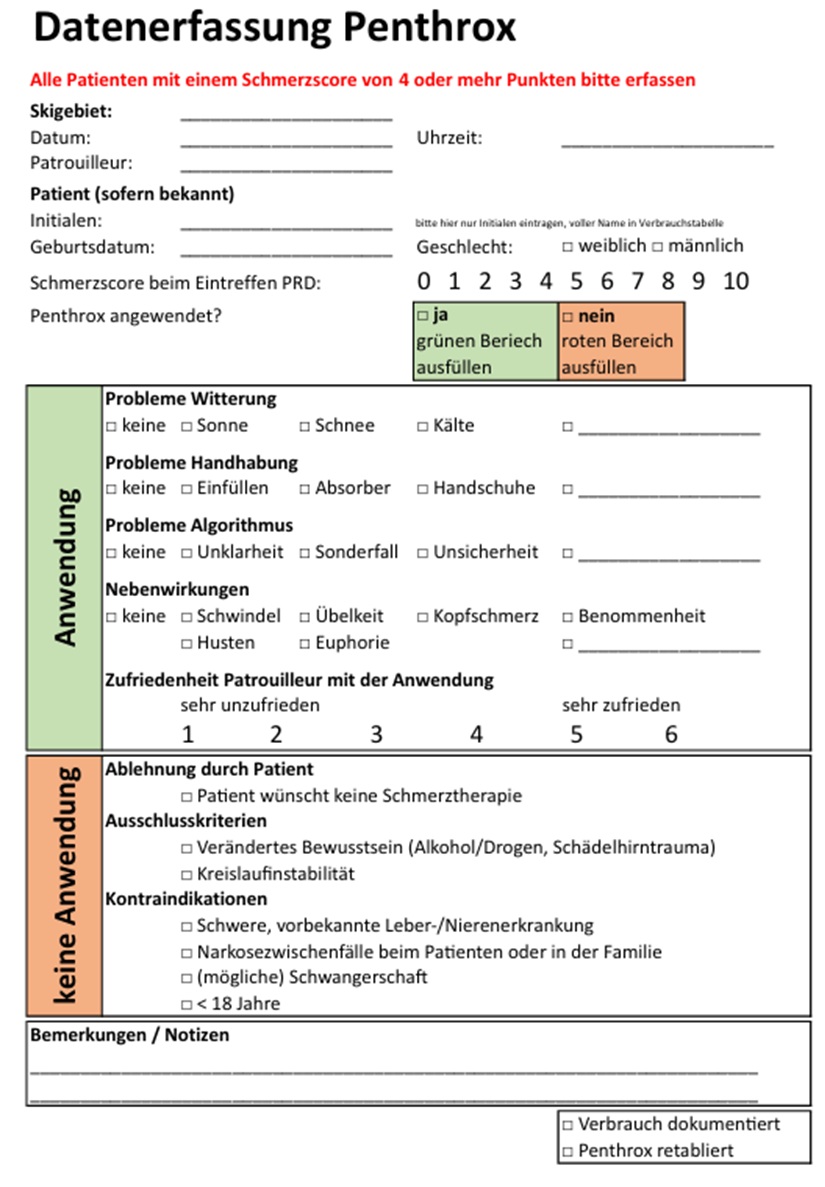

Supplement: Supplementary file 2 — Supplementary Material 2: Data collection form for the use of methoxyflurane in the participating ski areas. [file 13049_2024_1308_MOESM2_ESM.jpg]

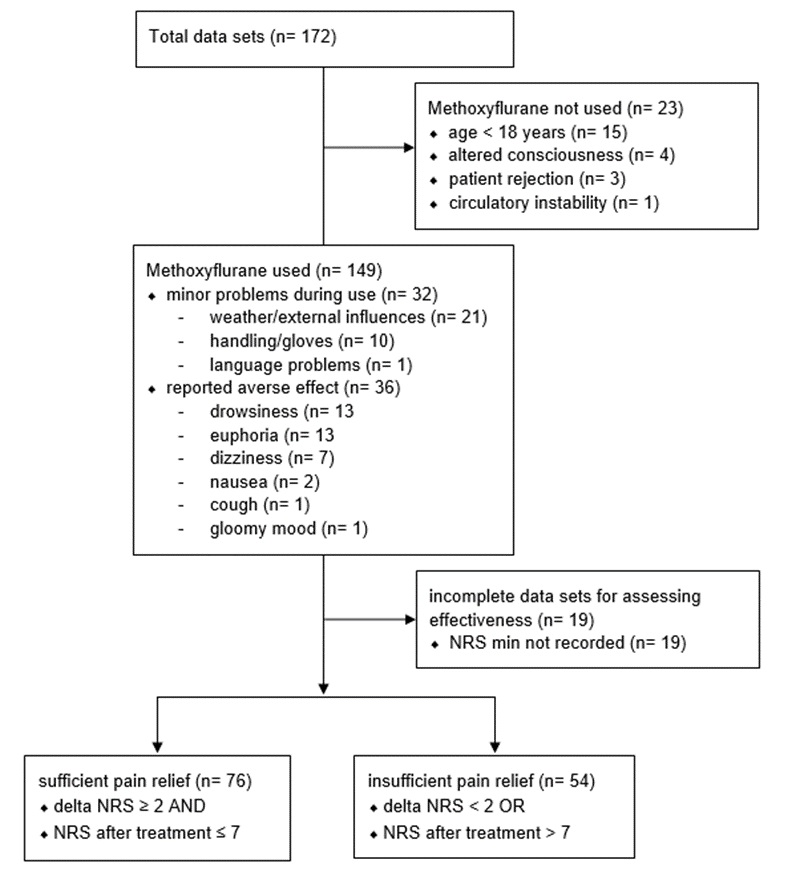

Supplement: Supplementary file 3 — Supplementary Material 3: Flow chart of patient distribution. [file 13049_2024_1308_MOESM3_ESM.jpg]
